# Identification of wheat stem rust resistance genes in wheat cultivars from Hebei province, China

**DOI:** 10.3389/fpls.2023.1156936

**Published:** 2023-03-30

**Authors:** Huiyan Sun, Ziye Wang, Rui Wang, Si Chen, Xinyu Ni, Fu Gao, Yazhao Zhang, Yiwei Xu, Xianxin Wu, Tianya Li

**Affiliations:** ^1^ College of Plant Protection, Shenyang Agricultural University, Shenyang, Liaoning, China; ^2^ Institute of Industrial Crops, Heilongjiang Academy of Agricultural Sciences, Harbin, China

**Keywords:** *Puccinia graminis* f. sp. *tritici*, wheat cultivars, resistance gene, wheat stem rust, *Sr* genes

## Abstract

Wheat stem rust is caused by *Puccinia graminis* f. sp. *tritici*. This major disease has been effectively controlled via resistance genes since the 1970s. The appearance and spread of new races of *P*. *graminis* f. sp. *tritici* (eg., Ug99, TKTTF, and TTRTF) have renewed the interest in identifying the resistance gene and breeding cultivars resistant to wheat stem rust. In this study, gene postulation, pedigree analysis, and molecular detection were used to determine the presence of stem rust resistance genes in 65 commercial wheat cultivars from Hebei Province. In addition, two predominant races 21C3CTHTM and 34MRGQM were used to evaluate the resistance of these cultivars at the adult-plant stage in 2021–2022. The results revealed that 6 *Sr* genes (namely, *Sr5*, *Sr17*, *Sr24*, *Sr31*, *Sr32*, *Sr38*, and *SrTmp*), either singly or in combination, were identified in 46 wheat cultivars. Overall, 37 wheat cultivars contained *Sr31*. *Sr5* and *Sr17* were present in 3 and 3 cultivars, respectively. Gao 5218 strong gluten, Jie 13-Ji 7369, and Kenong 1006 contained *Sr24*, *Sr32*, and *Sr38*, respectively. No wheat cultivar contained *Sr25* and *Sr26.* In total, 50 (76.9%) wheat cultivars were resistant to all tested races of *P*. *graminis* f. sp. *tritici* in field test in 2021–2022. This study is important for breeding wheat cultivars with resistance to stem rust.

## Introduction

Wheat stem rust, caused by *Puccinia graminis* Pers. f. sp. *tritici* Eriks. and E. Henn, is an ancient disease. During the 4th century BCE, the Romans considered April 25 every year as a festival to worship the rust god Robigus. They believed that this would prevent epidemics of cereal rusts by offering red cattle, foxes, and dogs to Robigus (Moscou and van Esse, 2017). The earliest record of the occurrence of wheat stem rust in China can be found in the agricultural book Ma Shou Nong Yan of Qing Dynasty in 1836, in which the disease is termed “jaundice” (Li and Zeng, 2002). This is a common wheat disease globally. Using the ecological niche-model of stem trust developed by Pardey et al. (2013), it was estimated that 66% (1.4 million km^2^) of the world’s wheat-cultivating region is climatically suitable for the development of the disease. Without durable stem-rust resistance, the average total global loss in wheat production is expected to be 306 million metric tons (MT) out of a total production of 23 billion MT (i.e., a loss of USD 54.7 billion when valued at the average wheat prices in 2010 in the United States) with a 90% chance (the 10th percentile) of losing at least 275 MT during 1918–1960 (Pardey et al., 2013). According to records, the widespread cultivation of a new spring wheat cultivar Marquis in Canada since 1912 led to the worst outbreak of wheat stem rust in the United States and Canada in 1916 (Peterson, 2018). In North Dakota, South Dakota, and Minnesota, the output yield loss in 1916 was 12.7, 10.3, and 9.4 bushels per acre, respectively (Peterson, 2018). On a larger scale, the loss from wheat stem rust was estimated at 200 million bushels in the United States (approximately 40% of crops in the United States) and 100 million bushels in Canada (Campbell and Long, 2001). Historically, several wheat stem rust epidemics have caused devastating losses in wheat yield in China (Wu and Huang, 1987; Lin et al., 2021). According to incomplete statistics, nine epidemics occurred in northeast spring wheat region from 1923 to 1964, the yield loss was 740 and 560 million kg in 1923 and 1948, respectively. In 1956 and 1958, the epidemic in the Jianghuai wheat area caused a wheat yield loss of 1 billion kg (Wu and Huang, 1987).

After the 1950s, wheat stem rust was effectively controlled via the combined effects of wide use of host resistance and eradicating common barberry (*Berberis vulgaris*; the alternate host of *Pgt*) in the United States and Europe (Rouse et al., 2012; Peterson, 2018). In the following five decades, except the major epidemic in Ethiopia in 1993 and 1994, wheat production in other regions in the world did not significantly suffer from wheat stem rust. The epidemic in Ethiopia caused significant yield loss of a popular wheat cultivar “Enkoy” (Shank, 1994; Singh et al., 2011). However, a new race of *P*. *graminis* f. sp. *tritici* (Isolate *Pgt*-Ug99) with virulence to *Sr31*, a widely present stem-rust resistance gene in wheat in the world, was first detected in Uganda in 1998 and designated as Ug99 (Pretorius et al., 2000). Based on the North American nomenclature system for naming *P*. *graminis* f. sp. *tritici* races, it was initially named TTKS and later redesignated as TTKSK (Wanyera et al., 2006; Jin et al., 2008). The Ug99 group of races (except for races TTKSF and TTKSP) is not only virulent to *Sr31* but also has the characteristics of rapid variation in the virulence (Singh et al., 2011). What is more worrying is that new pathogenic races are emerging with combined virulence to *Sr31* and some other resistance genes used in globally widely grown commercial wheat cultivars. For example, races TTKST, PTKST, TTHST, and TTKTT have combined virulence to *Sr31*, *Sr24*, and *Sr38* (Fetch et al., 2016; Nazari et al., 2021; Patpour et al., 2016), and race TTTSK has combined virulence to *Sr31*, *Sr36*, and *Sr38* (Pretorius et al., 2010). To date, 15 known variants of the Ug99 lineage have been identified, which are currently present in 14 countries. TKTTF, a new race of *P*. *graminis* f. sp. *tritici*, caused a severe wheat stem rust epidemic in 2013–2014 in the “Digalu” wheat cultivation area (approximately 20,000–40,000 ha) in southern Ethiopia, causing 100% yield loss in most wheat cultivation areas (Olivera et al., 2015). A new race TTRTF attacked thousands of hectares of durum wheat in 2016 in Sicily, Italy, causing an unusual epidemic of wheat stem rust in Europe since the 1950s (Bhattacharya, 2017). In recent years, other severe epidemic and outbreaks of wheat stem rust have been reported in Central Asia and the Caucasus region (Olivera Firpo et al., 2017; Olivera et al., 2022; Shamanin et al., 2019), and North Africa (Rust Tracker, 2021). These outbreaks of wheat stem rust indicate that the disease has now returned.

China is the world’s largest wheat producer and consumer, with wheat production playing a crucial role in national food security (Cao et al., 2019). Wheat is one of the most important crops in China, and flour food is very popular in northern China. Hebei Province, north China, is one of the major wheat-producing regions in the country and plays a significant role in the epidemiology and spread of wheat stem rust in China. According to the data of the National Bureau of Statistics (http://www.stats.gov.cn/), the annual wheat cultivation area in Hebei Province is approximately 2200 thousand ha, nearly one tenth of China’s wheat cultivation area. Historically, wheat stem rust occurred in this region in some years in past (Wu et al., 1963; Wu et al., 1966; Yao et al., 1997). Breeding disease-resistant wheat cultivars is the most economic, effective, and environmentally friendly strategy to control wheat stem rust (Hatta et al., 2021; Luo et al., 2022). Since the emergence of Ug99, we have increased our nationwide research on the variation in the virulence of *P*. *graminis* f. sp. *tritici*, identification of resistance genes, and resistance evaluation of cultivars to mitigate the threat posed by the Ug99 race group to Chinese wheat production (Cao et al., 2019; Li et al., 2016a; Xu et al., 2017; Li et al., 2021; Lin et al., 2021; Luo et al., 2022). However, in Hebei province, stem rust resistance hasn’t been a breeding goal. Therefore, the response of these cultivars to wheat stem rust in the field and the disease-resistance genes present in them are largely unknown. Therefore, in this study, we assessed the on-field infection response of wheat cultivars in Hebei province to two predominant races of *P*. *graminis* f. sp. *tritici* (21C3CTHTM and 34MRGQM) present in China. In addition, gene postulation, molecular testing, and pedigree analysis were performed to identify the genes resistant to wheat stem rust in these wheat cultivars.

## Materials and methods

### Plant materials and *P*. *graminis* f. sp. *tritici* races

A total of 65 wheat cultivars (**Table 1**) that were widely grown in Hebei province and 27 monogenic lines were included in this study (Jin et al., 2008). The 65 cultivars were provided by Dr. Hongfei Yan from College of Plant Protection, Hebei Agricultural University, Technological Innovation Center for Biological Control of Crop Diseases and Insect Pests of Hebei Province. The additional 27 monogenic lines include the 19 lines of the North American differential set (Jin et al., 2008) and eight additional lines (SwSr22T.B., Eagle, 73,214,3-1/9*LMPG, Mq(2)5XG2919, RL6082, RL6088, TAF2, and Fed/SrTt3) (Olivera Firpo et al., 2017; Olivera et al., 2022) with known *Sr* genes (**Table 2**) were preserved and propagated by the Institute of Plant Immunology of Shenyang Agricultural University Laboratory.

**Table 1:** Pedigree of tested cultivars, and stem rust infection response (IR) at the adult-plant stage.

No.	Cultivar	Pedigree	IRs at the adult-plant stage^a^			
			21C3CTHTM		34MRGQM	
			2021	2022	2021	2022
1	Henong 825	Linyuan 95-3019/Shi 4185	50R	10R	30R	2R
2	Henong 130-12	Not available	30MS	60MS	20R	5MR
3	Henong 6425	Henong 326/Chengyangdasui////Pin 39/Henongai 1xi//Tieganmai///Henong 326	30MR	10MR	5R	10R
4	Heng 95 Guan 26	Heng 84 Guan 749/Heng 87-263	0R	10R	30MR	10R
5	Henong 5290	Shan 160/Laizhou 95021	0R	10R	10R	30R
6	Henong 7069	Shan 160/Laizhou 95021	20MR	10R	20R	10R
7	Tainong 19	Laizhou 137/Jinan 17	0R	0R	0R	5R
8	Zhongmai 1	Yumai 18/80(6)-3-3-10/Northern west shorty (97-26)	0R	50MR	50S	80S
9	Luyuan 502	9940168/Jimai 19	20MR	10R	10R	10R
10	Xingmai 11	Jimai 36/Han 6172	10MR	10R	5R	5R
11	Xingmai 13	Heng 9117-2//Han 86-4032/85 zhong 47	40MR	50MR	20S	50MS
12	Zhongxin 5199	D703/Han 4589	5R	10R	50S	40S
13	Kenong 1006	Kenong 9204//Kenong 9204/Gaomai 5///9204R	0R	0R	0R	0R
14	Jimai 585	Taigu Male-sterile population	30R	20R	20R	20R
15	Shixin 811	Shixin 733/9731	50R	50R	5R	5R
16	Gaoyou 9409	8818/8901-11-14	0R	0R	10R	5R
17	Gaoyou 5766	030728/8901-11-14	10R	0R	0R	0R
18	Gaoyou 2018	9411/98172	0R	0R	30R	5R
19	Lunxuan 987	Aibai/Nongda 139/Beijin 837/T881	10MR	20MR	5R	10R
20	Yuanfeng 175	(92R149/Xian 87(30)) F1/Xiaoyan 6	30MR	40R	10R	5R
21	Changhan 85	Not available	5R	40R	0R	5R
22	Henong 822	88S522×92(698)	30R	20R	30R	20R
23	Henong 4198	Henong 326/#409//Henong 94342	30MS	30MS	0R	5R
24	Hanmai 9	Han 97-5040×89 Yuan 67	5R	10R	5R	10R
25	Hanmai 12	Han 93-4686/Henong 2458	5R	0R	0R	0R
26	Baomai 3	Changnong 621 ×Jinnong 192	0R	0R	0R	0R
27	Baomai 8	Linfen 857013×Changnong 921	20R	40R	0R	0R
28	Jinmai 61	Lumai 13/Lumai 14	40S	30MS	50MS	40MS
29	Gaoyou 503	78506/84s504	40R	10R	30R	10R
30	Kenong 2009	1BL-1RS Translocation lines 148/BE-1/Kn 1095//Kn 1095	0R	0R	40R	20R
31	Kenong 199	Shi 4185/Kn 9204	0R	0R	10R	5R
32	Yuanda 1	SA9219/Jimai 38	0R	0R	50MS	40MS
33	Henong 6331	Henong 6425/Jimai 38	20R	40R	30R	10R
34	Henong 6049	Shi 6021/Henong 91459	20R	10R	5R	5R
35	Kenong 9204	SA502×89-6021	20R	40R	0R	0R
36	Heng 7228	Ji 5418/Heng 5041	20R	10R	0R	0R
37	Shannong 17	L156/Liazhou 137	40MR	30R	0R	0R
38	Shannong 18	Lankao/924142	50MR	40MR	80S	80S
39	Heng 4399	Han 6172/Hengsui28	40R	50R	20R	30MR
40	Han 4589	Han 86-4032/85 Zhong 47	90S	80S	80S	70S
41	Liangxing 99	Ji 91102/Lumai 14//PH85-16	30R	30MR	20R	20R
42	Yingbo 700	Taigu Male-sterile/Ji93-5031	20R	30R	10R	10R
43	Jingdong 17	Jingdong 8/RHT3//931	30MR	5R	10R	10R
44	Shixin 616	Shixin 733/9831	20R	40R	0R	0R
45	Gaoyou 9908	8515-4×8901-11-14	0R	0R	50MS	40MS
46	Gaoyou 5218	Xinong 979/8901-11-14	40MS	40MS	10R	10R
47	Gao 5766 strong gluten	Not available	10R	30R	20R	5R
48	Hanmai 16	Han 5267/Han 4564	0R	0R	5R	5R
49	Zhongmai 155	Jimai19×Lumai21	20R	10R	20R	10R
50	Hanmai 17	Heng 84 Guan 749/Heng87-263/Gao 9411	0R	5R	50MS	40MS
51	Hengguan 35	Heng 84 Guan 749/Heng 87-263	30R	50R	0R	0R
52	Jie 36	Not available	0R	0R	0R	0R
53	Jie 22	Not available	5R	10R	0R	0R
54	Jie 15	Not available	20R	56R	5R	5R
55	Hanmai 15	Han 4564/Han 00-3207	10R	10R	20R	5R
56	Jie 13-Ji 7369	Not available	30MR	20MR	0R	0R
57	Shinong 086	Lumai 14 (C149)/F4530	30MR	20MR	0R	5R
58	Heng 4444	Xiaoyan 6×WM1	80S	60S	90S	70S
59	Hannong 1412	Liangxing 99/Pin 3	30MS	40S	50MS	50S
60	Shinong 956	Gaoyou 9415/Shixin 733	0R	0R	5R	5R
61	Shimai 22	Lin 8014/Jimai 38/Shi 4185	30R	20R	20R	5R
62	Shinong 952	Shixin 733/Gaoyou 9415	0R	0R	20R	10R
63	Shimai 18	92 Jian 3/T447/Jimai 38	5R	10R	0R	0R
64	Jie 29 Zhongxin 99	NA	20R	5R	0R	0R
65	Xinmai 296	935031/Lumai 23	50MS	40MS	0R	0R
66	Little club	–	90S	90S	80S	90S

a IRs in combination with severity were scored at the adult-plant stage in the field tests following the descriptions of Roelfs et al. (1992), where R, resistant; MR, moderately resistant; MS, moderately susceptible and S, susceptible.

**Table 2:** The infection types of 34 monogenic lines of wheat with known *Sr* genes inoculated with 10 *P. graminis* f. sp. *tritici*.

No.	Lines with known *Sr* genes	Infection types to *P*. *graminis* f. sp. *tritici* races^a^									
		R1^b^	R2	R3	R4	R5	R6	R7	R8	R9	R10
1	ISr5-Ra (*Sr5*)	1	1	1	3	3	4	4	3	4	4
2	CnS_T_mono_deri (*Sr21*)	2	1−	1	1−	1+	1−	1	2	1	1
3	Vernstine (*Sr9e)*	1−	;	1−	1	1	;	;	1+	;1	0
4	ISr7b-Ra (*Sr7b)*	4	3	4	4	4	3	3+	3	3	4
5	Lee (*Sr11*)	3	4	4	3	0	4	4	1	3+	4
6	ISr6-Ra (*Sr6*)	3	3+	3	3+	3	3+	3	3	3	3
7	ISr8a-Ra (*Sr8a*)	4	3+	3+	3	4	1	4	3	3	4
8	CnsSr9g (*Sr9g*)	4	4	3	4	3	4	3+	3	3+	4
9	CI12632/8*LMPG (*Sr36*)	0	4	4	1	0	0	1	0	0	4
10	W2691Sr9b (*Sr9b*)	3	4	3+	3	3	4	4	4	4	4
11	BtS30Wst (*Sr30*)	1	3	3+	;	1+	1−	1	;	1+	4
12	Prelude/8*Mq/2*/Esp 5/8/9 (*Sr17*)	4	3	3	;	0	;	;	1	;	1−
13	ISr9a-Ra (*Sr9a*)	4	3	3	4	3+	4	4	4	4	4
14	ISr9d-Ra (*Sr9d*)	3	4	4	3+	4	4	3	3	3+	4
15	W2691Sr10 (*Sr10*)	3	3	3	3	1+N	1	;	3−	3−	2
16	CnsSrTmp (*SrTmp*)	3−	1−	3N	;	0	1N	;	1	0	3
17	LcSr24Ag (*Sr24*)	3C	;1−	3	3	3	3−	4	3	3+	3
18	Sr31/6*LMPG (*Sr31*)	1−	;	1	;	1	;	;	1	1−	2
19	Trident (*Sr38*)	;	;	;	;	1	;	;	;	;1−	1
20	SwSr22T.B. (*Sr22*)	0	3−	1	0	1+	0	3+	0	2	2
21	Eagle (*Sr26*)	1	0	1	1+	1	1	2	1	1+	0
22	73,214,3-1/9*LMPG (*Sr27*)	1−	3+	3	3−	4	1+	0	4	1	3
23	Mq(2)5XG2919 (*Sr35*)	4	3−	;	1−	0	;	1+	;	1−	3
24	RL6082 (*Sr39*)	3	3−	3	3−	3−	3	3−	3	3+	3
25	RL6088 (*Sr40*)	1+	1	2	1	1+	0	0	2	1+	1
26	TAF 2 (*Sr44*)	1+	2	2	0	1+	0	1	2	0	1
27	Fed/SrTt3 (*SrTt3*)	1	1−	1+	2	2	;	1+	2	1+	2
28	Little club	4	3+	4	4	3+	4	4	4	4	4

a ITs scored in the greenhouse seedling tests were based on a 0−4 scale (Stakman et al., 1962), where ITs 0, 1, or 2 were considered resistant and ITs 3 or 4 were considered susceptible, and symbols + and – indicated slightly larger and smaller pustule sizes, respectively.

b R1−R10 represent the tested races 21C3CTHTM, 21C3CTTSC, 21C3CTTTM, 34MTGSM, 34MKGQM, 34MRGQM, 34C3MKGSM, 34C3MTGQM, 34C6MTGSM, and mutant strain.

The races of *P*. *graminis* f. sp. *tritici* used in this study were 21C3CTHTM, 21C3CTTTM, 21C3CTTSC, 34MKGQM, 34MTGSM, 34MRGQM, 34C3MTGQM, 34C3MKGSM, 34C6MTGSM, and a mutant strain 34MTSRM obtained via artificial irradiation of ultraviolet light. These *P*. *graminis* f. sp. *tritici* races were named as per the North American nomenclature system (Jin et al., 2008; Roelfs and Martens, 1988) and a new Chinese compound differential system for *P*. *graminis* f. sp. *tritici* (Cao et al., 2019).

### Field tests

In the third week of March 2021 and 2022, all wheat cultivars were planted in the experimental site of Plant Protection College of Shenyang Agricultural University. The cultivation method was to plant one row of each cultivar (length 1 m and row spacing 25 cm) and one row of Little club (susceptibility check cultivar) for every 10 rows. 21C3CTHTM and 34MRGQM, the races of *P*. *graminis* f. sp. *tritici* dominant in recent two decades in China, were used for the field test. Inoculation was conducted at the jointing stage according to the method by Wu et al. (2020). Maximum severity and infection response (IR) were scored approximately 25 days after inoculation using a modified Cobb scale as described by Roelfs et al. (1992) when Little club exhibited disease severity of 90%–100%. IRs included I for immune, R for resistant, MR for moderately resistant, MS for moderately susceptible, and S for susceptible.

### Seedling testing

The 10 races of *P*. *graminis* f. sp. *tritici* were used to inoculate the wheat cultivars, 27 monogenic lines with known *Sr* genes, and Little club (cultivar for susceptibility check). In total, 8–10 seeds of each genotype were planted in porcelain pots with a diameter of 12 cm in the greenhouse and inoculated with each *Pgt* race. At the two-leaf stage (one leaf and one sprout), the seedlings were inoculated with each race. Briefly, the leaves were sprayed with 0.05% Tween-20 aqueous solution, dusted with urediniospores mixed with talcum at a ratio of 1:20 (vol/vol), and sprayed again with 0.05% Tween-20 aqueous solution to produce a moisturizing film. The inoculated plants were kept moist in dark at 18–20°C for 16 h and further cultured in a glass greenhouse at 20 ± 1°C. After 2 weeks of inoculation, the infection types (ITs) were recorded based on the 0–4 scale described by Stakman et al. (1962). The ITs 0,;, 1−, 1, 1+, 2, and 2+ were classified as low ITs (resistant reaction), and 3−, 3, 3+, and 4 were classified as high ITs (susceptible reaction). According to the ITs of the cultivar evaluated and those of the wheat lines with known *Sr* gene, gene postulation was conducted as per the principle proposed by Dubin et al. (1989).

### Molecular marker analysis

Genomic DNA of 65 wheat cultivars was extracted using cetyl trimethyl ammonium bromide (CTAB) (Xu et al., 2018). All cultivars were evaluated with molecular markers closely linked to six known genes *Sr31*, *Sr38*, *Sr32*, *Sr24*, *Sr25*, and *Sr26*. Polymerase chain reaction (PCR)-specific primers were synthesized by Shanghai Biotech Co., Ltd, China (**Supplementary Table 1**). PCR amplification was conducted in 25-µL reaction mixture, which included 1 µL of 10 µmol L^−1^ of each primer, 0.5 µL of 10 mmol L^−1^ deoxyribonucleoside triphosphates, 2.5 µL of 10× buffer (Mg^2+^), 0.2 µL of 5 U µL^−1^ taq polymerase, and 2 µL of 40 ng µL^−1^ DNA. The volume was made up to 25 µL using deionized water. The annealing temperature, allele size, and primer sequences for PCR are given in **Supplementary Table 1**.

## Results

### Field evaluation

The IRs of 65 wheat cultivars were tested with two predominant races 21C3CTHTM and 34MRGQM during 2021 and 2022 (**Table 1**). The IRs of all wheat cultivars could be classified into three groups immune, resistance to moderately resistance, and moderately susceptible to susceptible (**Table 3**). 56 (86.2%) and 55 (84.6%) wheat cultivars were resistant to the races 21C3CTHTM and 34MRGQM, respectively, in the field tests in 2021 and 2022. Overall, 50 (76.9%) wheat cultivars were resistant to all tested races.

**Table 3:** Resistance and susceptibility of 65 wheat cultivars to 2 races of *P. graminis* f. sp. *tritici* at the at adult-plant stage in 2021 and 2022.

Races	Immune		Resistance-moderately resistance		Moderately susceptible-susceptible	
	2021	2022	2021	2022	2021	2022
21C3CTHTM	17 (26.2)^a^	15 (23.1)	39 (60.0)	41 (63.1)	9 (13.8)	9 (13.8)
34MRGQM	20 (30.8)	16 (24.6)	35 (53.8)	39 (61.0)	10 (15.4)	10 (15.4)
All races	3 (4.6)	5 (7.7)	47 (72.3)	45 (69.2)	15 (23.1)	15 (23.1)

a 17 (26.2): 17= Number of wheat lines immune to tested race, 26.2 = Percentage of immune wheat lines in total tested lines.

### *Sr* genes in the wheat cultivars based on gene postulation and molecular marker analysis

The monogenic lines with known *Sr* genes (*Sr6*, *Sr7b*, *Sr9a*, *Sr9b*, *Sr9d*, *Sr9g*, and *Sr39*) and Little club exhibited high ITs to all *Pgt* races. In contrast, 9 *Sr* genes (*Sr9e*, *Sr21*, *Sr31*, *Sr38*, *Sr26*, *Sr40*, *Sr44*, and *SrTt3*) exhibited low ITs to all races. Therefore, these 18 *Sr* genes could not be deduced by gene postulation. However, 13 monogenic wheat lines (each containing *Sr5*, *Sr11*, *Sr8a*, *Sr36*, *Sr30*, *Sr17*, *Sr10*, *SrTmp*, *Sr24*, *Sr22*, *Sr27*, *Sr32*, and *Sr35*) exhibited high and low ITs for each of the 10 tested *Pgt* races; therefore, these genes could be used for gene postulation (**Table 2**). Molecular detection and gene postulation identified 6 *Sr* genes (*Sr5*, *Sr17*, *Sr24*, *Sr31*, *Sr32*, *Sr38*, and *SrTmp*), either singly or in combination, in 46 wheat cultivars.

**Figure 1:**
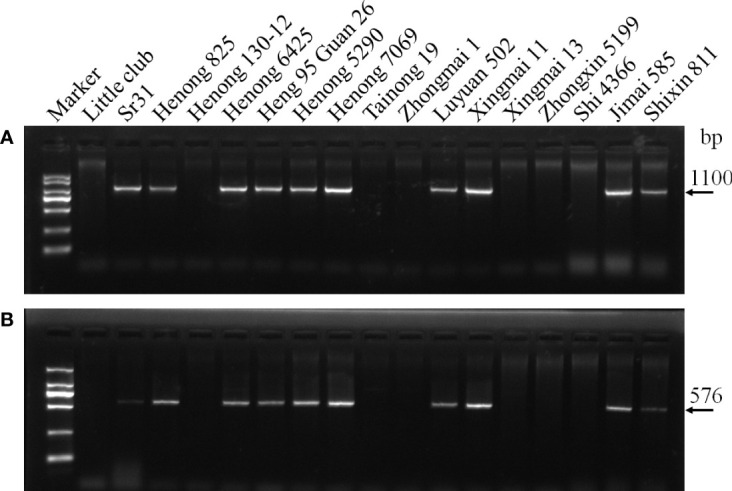
Amplification result for parts of wheat cultivars with markers SCSS30.2_576_ and Iag95. **(A)** Iag95, **(B)** SCSS30.2_576_.

The molecular marker analysis revealed that 37 wheat cultivars carried *Sr31* (**Table 4**; **Figure 1**). All the lines that tested positive for the *Sr31* marker were also resistant to all the evaluated races. None of these races are virulent to *Sr31* gene, this again confirmed that *Sr31* is present in these cultivars. Three wheat cultivars (Zhongmai 1, Xingmai 13, and Hanmai 17) were postulated to be carrying *Sr5*. Zhongmai 1 and Xingmai 13 contained only *Sr5*; these cultivars were resistant to the three races avirulent to *Sr5* and were susceptible to 7 races virulent to *Sr5* (34MTGSM, 34MKGQM, 34MRGQM, 34C3MKGSM, 34C3MTGQM, 34C6MTGSM, and mutant strain). Hanmai 17 was susceptible to races 34MTGSM and 34MKGQM but resistant to three *Sr5-*avirulent races (21C3CTHTM, 21C3CTTSC, and 21C3CTTTM) and some other races, indicating that this cultivar contains *Sr5* and other unknown *Sr* gene(s). Three wheat cultivars, namely, Henong 4198, Gao 5218 strong gluten, and Jie 22, might contain *Sr17* because they were resistant to all *Sr17*-avirulent races (34MTGSM, 34MKGQM, 34MRGQM, 34C3MKGSM, 34C3MTGQM, 34C6MTGSM, and mutant strain) and susceptible to three *Sr17-*virulent races. Although cultivar Heng 4444 exhibited a resistant/susceptible profile that indicates the presence of *Sr17* at the seedling stage, in field evaluations against *Sr17*-avirulent race 34MRGQM exhibited a S response, indicating that *Sr17* may not be present in this cultivar. Moreover, Gao 5218 strong gluten was confirmed to contain *Sr24* using a specific molecular marker. Two wheat cultivars, namely, Jinmai 61 and Shannong 18, displayed low ITs to three races (21C3CTHTM, 21C3CTTTM, and mutant strain) and high ITs to the remaining 7 races (34C6MTGSM, 34MTGSM, 34MKGQM, 34MRGQM, 34C3MKGSM, 34C3MTGQM, and 21C3CTTSC). This was consistent with the ITs of *SrTmp*, indicating that they might contain this gene. However, Jinmai 61 exhibited 30MS to 40S susceptible response to race 21C3CTHTM in field tests, therefore, indicating that this cultivar does not carry *SrTmp*. Two wheat cultivars Jie 13-Ji 7369 and Kenong 1006 contained *Sr32* and *Sr38*, respectively, as revealed by molecular marker analysis. Four wheat cultivars Lunxuan 987, Kenong 199, Heng 4399, and Shimai 22 exhibited resistance to all the races at the seedling stage and an MR to R infection response at the adult-plant stage, this indicated that these cultivars might contain one or more *Sr* genes (*Sr9e*, *Sr21*, *Sr31*, *Sr38*, *Sr26*, *Sr40*, *Sr44*, and *SrTt3*) that had low ITs to all races, as well as unknown effective resistance genes.

**Table 4:** Seedling infection types and presence or absence of stem rust resistance (*Sr*) genes in 65 wheat cultivars based on gene postulation using 10 P. *graminis* f. sp. *tritici* and molecular markers.

No.	Cultivar	*Sr* gene	Infection types to *P*. *graminis* f. sp. *tritici* races^a^									
			R1^b^	R2	R3	R4	R5	R6	R7	R8	R9	R10
1	Henong 825	*Sr31* ^d^	1+	0	1	;	1+	1	1	0	1−	2
2	Henong 130-12	*-*	3	;	4	4	2	3+	3+	3−	2	2
3	Henong 6425	*Sr31* ^d^	1	0	1+	;	1	1+	1	0	+	2
4	Heng 95 Guan 26	*Sr31* ^d^	1+	1+	0	0	1+	1	1	0	1	1
5	Henong 5290	*Sr31* ^d^	0	0	;	0	1	1	0	0	1	1+
6	Henong 7069	*Sr31* ^d^	1	1−	0	;	1	;	1	0	;	1+
7	Tainong 19	*-*	0	0	3	0	;	3−	4	0	2	3
8	Zhongmai 1	*Sr5* ^c^	1	0	1+	3	3	4	4	3−	3−	3−
9	Luyuan 502	*Sr31* ^d^	1	0	1+	0	2	1	1	0	1	1
10	Xingmai 11	*Sr31* ^d^	;	0	1+	;	1+	1+	1+	;	1−	1+
11	Xingmai 13	*Sr5* ^c^	;	1+	2	3	3+	4	4	4	3+	3
12	Zhongxin 5199	*-*	1	3	2	0	3	4	4	;	;	3
13	Kenong 1006	*Sr38* ^d^	1	0	;	0	1	;	2	0	1+	;
14	Jimai 585	*Sr31* ^d^	1	0	1	0	1+	1+	;	0	1	2
15	Shixin 811	*Sr31* ^d^	1	0	1	0	;	1	1	0	1+	2
16	Gaoyou 9409	*Sr31* ^d^	0	0	1	;	1	1	1	0	1−	2
17	Gaoyou 5766	*Sr31* ^d^	1	;	1	1	1	1	1	0	;	2
18	Gaoyou 2018	*-*	0	0	4	0	3−	2	3+	1	2	3+
19	Lunxuan 987	*-*	1+	0	;	0	1	2	;	0	0	1
20	Yuanfeng 175	*Sr31* ^c,d^	1+	0	1	0	1+	;	1+	0	;	2
21	Changhan 85	*Sr31* ^c,d^	;	0	1	1	1−	;	1	1	1	2
22	Henong 822	*Sr31* ^d^	;1−	0	;	0	1	;	1	1	;	1
23	Henong 4198	*Sr17* ^c^	3	3	3	0	0	1	−	0	1−	1
24	Hanmai 9	*Sr31* ^d^	1	1	1	0	1	;	1	0	0	1
25	Hanmai 12	*Sr31* ^d^	1	0	1	0	0	;	1	0	1−	1
26	Baomai 3	*Sr31* ^d^	0	0	;	0	0	0	0	;	1−	2
27	Baomai 8	*-*	2	3−	3	1	0	2	1+	4	1	4
28	Jinmai 61	*-*	3	1+	4	;	1+	2	1+	0	1	3
29	Gaoyou 503	*Sr31* ^d^	;	0	0	0	1	;	;	0	0	1
30	Kenong 2009	*Sr31* ^d^	0	0	1	0	1	0	1−	0	1	1+
31	Kenong 199	*-*	0	;	0	0	1	2	1+	0	1−	1+
32	Yuanda 1	*-*	0	3	3	0	3	;	0	0	;	4
33	Henong 6331	*Sr31* ^d^	1	0	0	1	2	;	1+	0	1+	2−
34	Henong 6049	*Sr31* ^d^	1	;	0	0	0	0	0	0	0	2
35	Kenong 9204	*Sr31* ^d^	1	;	0	;	0	1	1	0	0	1
36	Heng 7228	*Sr31* ^d^	1	0		0	0	1	;	0	1	2
37	Shannong 17	*Sr31* ^d^	1+	0	1	0	;	0	;	0	1	1+
38	Shannong 18	*SrTmp*	3	0	3	0	1	;	2	0	1	3
39	Heng 4399	*-*	1+	0	1	0	2	1	2	0	2	1+
40	Han 4589	*-*	3	0	2	0	3	3	2	0	2	2
41	Liangxing 99	*Sr31* ^d^	1+	0	1+	0	1	1	1+	1	0	2
42	Yingbo 700	*Sr31* ^d^	1+	1+	;	;	;	1	1+	1	;	2
43	Jingdong 17	*Sr31* ^d^	1+	1−	1	0	;	1+	1	0	0	2
44	Shixin 616	*Sr31* ^d^	1	1−	1	0	0	;	0	0	0	;
45	Gaoyou 9908	*-*	0	0	2	0	3	4	2	0	4	2
46	Gao 5218 strong gluten	*Sr17* ^c^, *Sr24* ^d^	3	2	3	0	1	;	2	0	0	1
47	Gao 5766 strong gluten	*-*	1	4	4	0	2	4	3	4	4	3
48	Hanmai 16	*Sr31* ^d^	0	0	0	0	;	1+	1	0	1	;
49	Zhongmai 155	*Sr31* ^d^	1+	2	1+	1	1+	0	0	0	1	2
50	Hanmai 17	*Sr5 +*?	0	0	;	3	3	0	;	0	1	1
51	Hengguan 35	*Sr31* ^d^	;	0	2	0	0	1+	1	1	1	2
52	Jie 36	*Sr31* ^d^	0	0	1+	0	0	1+	1	0	1+	2
53	Jie 22	*Sr17* ^c^	4	4	3	0	0	1+	1	0	2	1
54	Jie 15	*Sr31* ^d^	1	0	1−	0	1−	1	1	1	0	1+
55	Hanmai 15	*Sr31* ^d^	1	0	1+	0	1+	1	1	0	0	1+
56	Jie 13-Ji 7369	*Sr32* ^d^	3	1−	3−	3−	0	0	3	3	0	1
57	Shinong 086	*-*	2	0	3	0	1−	3−	3	0	3	2
58	Heng 4444	*-*	4	3	4	0	1	1	1+	1	1+	1+
59	Hannong 1412	*-*	3	0	3	0	3	4	3−	1−	3+	2
60	Shinong 956	*Sr31* ^d^	0	0	1	0	;	1	1	;	;	1
61	Shimai 22	*-*	2	1	1+	0	1	1+	1+	1+	2	1
62	Shinong 952	*-*	0	4	0	3	1	1	3−	2	0	1+
63	Shimai 18	*Sr31* ^d^	1	0	1−	0	0	;	1+	1	1−	1
64	Jie 29 Zhongxin 99	*Sr31* ^d^	1	0	;	1+	0	1	0	0	1	;
65	Xinmai 296	*-*	3	0	2	0	0	4	3+	;	3+	1
66	Little club	*-*	4	3+	4	4	3+	4	4	4	4	4

a ITs scored in the greenhouse seedling tests were based on a 0−4 scale (Stakman et al., 1962), where ITs 0, 1, or 2 were considered resistant and ITs 3 or 4 were considered susceptible; and symbols + and – indicated slightly larger and smaller pustule sizes, respectively.

b R1−R10 represent the tested races 21C3CTHTM, 21C3CTTSC, 21C3CTTTM, 34MTGSM, 34MKGQM, 34MRGQM, 34C3MKGSM, 34C3MTGQM, 34C6MTGSM, and mutant strain.

c Postulation of *Sr* genes based on gene postulation.

d Detection of *Sr* genes based on molecular marker.

## Discussion

In the past two decades, with the emergence of new races of *P*. *graminis* f. sp. *tritici*, such as Ug99, TKTTF, and TTRTF, wheat stem rust is posing a serious threat to wheat production worldwide (Pretorius et al., 2000; Olivera et al., 2015; Singh et al., 2015; Bhattacharya, 2017; Wu et al., 2020). To mitigate this threat, intensive global efforts are underway to carefully monitor the evolution and migration of races, identify the pathotypes of the races, screen disease-resistant germplasms, and breed wheat cultivars with for durable disease resistance (Singh et al., 2011; Moscou and van Esse, 2017; Cao et al., 2019). Therefore, in this study, 65 wheat cultivars in Hebei Province, which played a bridge role in the epidemiology of wheat stem rust in China, were evaluated for the resistance to wheat stem rust in the field. Gene postulation and molecular marker analysis were conducted to identify the *Sr* genes present in these cultivars. Overall, 37 wheat cultivars (accounting for 56.9% of the tested cultivars) carrying *Sr31* were identified, and these wheat cultivars were resistant to all races in the 2-year field test. Our results are consistent with many previous studies, proving that most Chinese wheat cultivars may contain *Sr31* (Li et al., 2016a). This is mainly due to 1BL/1RS translocation (e.g., in wheat lines such as Alondra S, Aftab LeEr, Avorora, Kavkaz, Lovrin 10, Lovrin 13, and Neuzucht) carrying the linkage gene *Sr31*-*Lr26*-*Yr9*-*Pm8*, which has been widely used in disease-resistant breeding programs in China since the 1960s. Although Ug99 is virulent to *Sr31*, no race virulent to this gene has been found in China (Li et al., 2018). Overall, 37 wheat cultivars with *Sr31* as revealed molecular detection exhibited low ITs to 10 races tested at the seedling stage and to 21C3CTHTM and 34MRGQM in the field test. Further pedigree analysis confirmed that the presence of *Sr31* in Henong 825 and Jingdong 17 can be derived from Lovrin 10. 1BL/1RS translation was observed in the pedigrees of Henong 5290, Henong 7069, and Kenong 2009 wheat cultivars. *Sr31* in Henong 6049, Shimai 18, and Shimai 22 might be from Avorara or Lovrin 10 because these *Sr31* donors were present in the pedigree of these three wheat cultivars. Lovlin 13 and Lumai 14 containing *Sr31* were found in the pedigree of Luyuan 205, Zhongmai 155, Liangxing 99, Shannong 17, and Yingbo 700 wheat cultivars, indicating that the presence of *Sr31* in these cultivars may be inherited from Lovlin 13 or Lumai 14. Through molecular detection and gene postulation, Gebrewahid et al. (2020) confirmed that Henong 6049, Shimai 18, and Shixin 616 contain *Lr26*. Because *Lr26* and *Sr31* are closely linked, this result supported our observations.

*Sr5*, located on chromosome 6D, is present in a standard differential Reliance of *P*. *graminis* f. sp. *tritici* (Sears et al., 1957; Stakman et al., 1962). This gene is widely distributed in global wheat cultivars such as Cheyenne, Chisholm, Justin, Winoka, Arthur, Fortuna, Centurk, Kitt, Thatcher, and Agatha in North America; Catcher, Bindawarra, Aroona, and Avocet in Canada; and Panis, Primus, and Orlando in Europe (McIntosh et al., 1995). *Sr5* is the second most commonly used wheat stem rust resistance gene in China after *Sr31* and confers valuable resistance against the 21C3 race group of *P*. *graminis* f. sp. *tritici* (Cao et al., 2019). This is mainly because wheat cultivars Minn 2761, Dongfanghong 2, Kefang 1, Dongxie 3, Alondra’S’, Orofen, and their derivatives containing *Sr5* have been grown for a long time and continually used in breeding programs (Wu et al., 2020). In this study, Zhongmai 1, Xingmai 13, and Hanmai 17 wheat cultivars were postulated to contain *Sr5*. Pedigree analysis revealed that the parent Yumai 18 of Zhongmai 1 contained rust-resistant germplasm St2422/464 from Italy (Yang et al., 2009). Therefore, it is speculated that *Sr5* contained in Zhongmai 1 may originate from St2422/464.

*Sr17*, originating from *Triticum turgidum* var. dicoccum cv. Yaroslav emmer and located on 7BL of the wheat chromosome, is very common in Chinese wheat cultivars and provides resistance to all *Sr5*-virulent races and some races of the *Sr5*-avirulence group in China (Cao et al., 2019). Moreover, *Sr17* is present in many wheat cultivars from the United States, Canada, India, and Mexico, particularly in those *Lr14a* and *Pm5* as all three genes are linked (Roelfs and Mcvey, 1979). *Sr17* is temperature sensitive. It is more effective against *P*. *graminis* f. sp. *tritici* at low temperature. When the temperature is higher than 25°C, the resistance becomes ineffective. In this study, Henong 4198, Gao 5218 strong gluten, and Jie 22 were postulated to contain this gene, and Gao 5218 strong gluten was confirmed to contain *Sr24* using a specific molecular marker. *Sr24*, derived from *Thinopyrum ponticum*, is completely associated with *Lr24*. This gene is widely used in global wheat disease resistance breeding programs. Virulent races to *Sr24* has been reported in South Africa, Kenya, Mozambique, India, and other countries; however, in North America, it is rarely reported (Jin et al., 2008; Xu et al., 2018). In China, the monogenic wheat line LcSr24Ag (carring *Sr24*) was first introduced from Cereal Disease Laboratory in University of Minnesota in 1990. When the virulence frequency test was conducted in the same year, 54 of the 89 tested *P. graminis* f. sp. *tritici* isolateswere virulent to the *Sr24* (Yao et al., 1993). Since then, many *P. graminis* f. sp. *tritici* isolates with virulence to *Sr24* have been detected, and the virulence frequency is approximately 50% (Wu and Huang, 1987; Cao et al., 2019). Considering the resistance of this gene to new races TKTTF and TTRTF, which caused epidemics in Ethiopia and Italy in 2014 and 2016, respectively (Olivera et al., 2019), we conducted molecular detection using 371 dominant cultivated wheat cultivars (including 65 in this study) in northern China using the molecular marker Sr24 # 12; only 1 wheat cultivar Gao 5218 strong gluten (in this study) may contain this gene. This is consistent with the study by Zhang (2015); screening using molecular markers linked to *Lr24* revealed that none of the 406 wheat cultivars included in their study carried this gene (Zhang, 2015). These results revealed that only few wheat cultivars in China contain this gene.

*SrTmp*, originated from *Triumph*, was located on chromosome arm 6DS in a region similar to *SrCad* and *Sr42* (Hiebert et al., 2016). This gene was present in commercial cultivars of hard red winter wheat to some extent since 1874 when a wheat cultivar Turkey was introduced (Roelfs and Mcvey, 1979). This cultivar is particularly widely used in the United States, Pakistan, Ethiopia, Nebraska, and other countries (Sajid et al., 2009; Cao et al., 2019). This gene not only has good resistance to many races of *P*. *graminis* f. sp. *tritici* in China but also has resistance to Ug99 and some Ug99 variants (Cao et al., 2019; Li et al., 2016b). In present study, Shannong 18 was postulated to carry *SrTmp*. Pedigree tracing analysis revealed that Shannong 18 is derived from Yannong 74. Youbaomai, Yannong 74’s parent, contains leaf-rust resistance genes *Lr1* and *Lr35* and stem-rust resistance gene *SrTmp*. Therefore, it is speculated that *SrTmp* contained in these two cultivars comes from Youbaomai (Zhuang, 2003).

Genes *Sr25*, *Sr26*, and *Sr32* confer resistance to all *P*. *graminis* f. sp. *tritici* races belonging to the Ug99 lineage. Considering the disastrous threat posed by the Ug99 race group on world food security, Nobel Peace Prize laureate Norman Borlaug called for a coordinated global campaign to monitor the populations of Ug99 races and screen wheat germplasms for resistance to Ug99. This can help to control wheat stem rust epidemics and mitigate the potential impact on food security (Singh et al., 2011; Wu et al., 2020; Lin et al., 2021). In view of this, we used molecular markers closely linked to *Sr25*, *Sr26*, and *Sr32* to screen these genes to identify possible resistant germplasm to Ug99. To date, of the 891 wheat cultivars tested by us, only 1, 2, and 2 wheat cultivars contained *Sr25*, *Sr26*, and *Sr32*, respectively (including 1 in this study). Therefore, very few wheat cultivars from China contain these three genes. *Sr38*, originated from *T*. *ventricosum*, is completely linked with leaf-rust resistance gene *Lr37* and stripe rust gene *Yr17.* Because of the combined resistance to all three rust diseases, *Sr38* was widely used in disease-resistance wheat breeding in China (Wu et al., 2020). However, surprisingly, only one of the 65 main wheat cultivars tested in this study possibly contained this gene. Although *Sr38* has become ineffective for Ug99 and its variants, no race of *P*. *graminis* f. sp. *tritici* with virulence to this gene has been detected in China.

In summary, our study demonstrated that the wheat cultivars in Hebei Province have very limited genes (most abundant being *Sr31*) that are resistance to stem rust. These cultivars currently exhibit good resistance to wheat stem rust in Hebei Province. However, if the virulence of *P. graminis* f. sp. *tritici* changes in future, particularly once Ug99 race group spreads to China, it will lead to serious epidemics and huge wheat yield losses. Therefore, more disease-resistance genes should be used in disease-resistance breeding to maintain the durable resistance of wheat cultivars.

## Data availability statement

The original contributions presented in the study are included in the article/**Supplementary Material**. Further inquiries can be directed to the corresponding author.

## Author contributions

TL conceived and designed the experiments, analyzed the data, authored or reviewed drafts of the paper, approved the final draft. HS, XN, RW and ZW performed the experiments, authored or reviewed drafts of the paper. SC, FG and YZ prepared figures and/or tables. XW and YX analyzed the data, prepared figures and/or tables. All authors contributed to the article and approved the submitted version.
