# Supplementary material for: Identification of wheat stem rust resistance genes in wheat cultivars from Hebei province, China
Source: Front Plant Sci. 2023 Mar 30;14:1156936. doi: 10.3389/fpls.2023.1156936 (PMC10098322; doi:10.3389/fpls.2023.1156936)
Supplement: Supplementary file 1 [file Table_1.doc]

Supplementary Table 1 PCR primers and their conditions for detecting *Sr* genes as well as specific fragment sizes.

| Gene | Primer | Sequence of primer (5´→3´) | Annealng temp. (°C) | Size (bp) | Reference |
| --- | --- | --- | --- | --- | --- |
| *Sr31* | SCSS30.2_576_ | GTCCGACAATACGAACGATT | 60 | 576 | Das et al., 2006 |
|  | SCSS30.2_576_ | CCGACAATACGAACGCCTTG |  |  |  |
|  | Iag95 | CTCTGTGGATAGTTACTTGATCGA | 55 | 1100 | Mago et al., 2002 |
|  | Iag95 | CCTAGAACATGCATGGCTGTTACA |  |  |  |
| *Sr38* | VENTRIUP–LN2 | GGGGCTACTGACCAAGGCT | 65 | 259 | Helguera et al., 2003 |
|  | VENTRIUP–LN2 | TGCAGCTACAGCAGTATGTACACAAAA |  |  |  |
| *Sr32* | csSr32#2 | CAAATGAATAGAAAAACCCGTGCT | 60 | 152 | Mago et al., 2013 |
|  | csSr32#2 | CACACACTGTTTTCCGTTGC |  |  |  |
| *Sr24* | Sr24#12 | CACCCGTGACATGCTCGTA | 59 | 500 | Mago et al., 2005 |
|  | Sr24#12 | AACAGGAAATGAGCAACGATGT |  |  |  |
| *Sr25* | Gb | CATCCTTGGGGACCTC | 50 | 191 | Liu et al., 2010 |
|  | Gb | CCAGCTCGCATACATCCA |  |  |  |
| *Sr26* | Sr26#43 | AATCGTCCACATTGGCTTCT | 60 | 207 | Mago et al., 2005 |
|  | Sr26#43 | CGCAACAAAATCATGCACTA |  |  |  |
